# Risk factors for death from other diseases after curative gastrectomy and lymph node dissection for gastric cancer

**DOI:** 10.1186/s12893-024-02313-6

**Published:** 2024-01-08

**Authors:** Takaaki Hanyu, Hiroshi Ichikawa, Yosuke Kano, Takashi Ishikawa, Yusuke Muneoka, Yuki Hirose, Kohei Miura, Yosuke Tajima, Yoshifumi Shimada, Jun Sakata, Toshifumi Wakai

**Affiliations:** 1 Division of Digestive and General Surgery, Niigata University Graduate School of Medical and Dental Sciences, 1-757 Asahimachi-dori, Chuou-ku, Niigata, 951-8510 Japan; 2 Department of Surgery, Shibata Prefectural Hospital, 1-2-8 Hon-cho, Shibata, Niigata 957- 8588 Japan

**Keywords:** Gastric cancer, Gastrectomy, Death from other diseases, Risk factors, Comorbidity, Total gastrectomy, Postoperative complications

## Abstract

**Background:**

Recent advances in treatment are expected to bring a cure to more patients with gastric cancer (GC). Focusing on the risk of death from other diseases (DOD) has become a crucial issue in patients cured of GC. The aim of this study was to elucidate the risk factors for DOD in patients who underwent curative gastrectomy with lymph node dissection for GC.

**Methods:**

We enrolled 810 patients who underwent curative gastrectomy with lymph node dissection for GC from January 1990 to December 2014 and had no recurrence or death of GC until December 2019. We investigated the risk factors for DOD defined as death excluding death from a malignant neoplasm, accident, or suicide after gastrectomy, focusing on the perioperative characteristics at gastrectomy.

**Results:**

Among 315 deaths from any cause, 210 died from diseases other than malignancy, accidents and suicide. The leading cause of DOD was pneumonia in 54 patients (25.7%). The actual survival period in 167 patients (79.5%) with DOD was shorter than their estimated life expectancy at gastrectomy. Multivariate analysis revealed that a high Charlson Comorbidity Index score (score 1–2: hazard ratio [HR] 2.192, 95% confidence interval [CI] 1.713–2.804, *P* < 0.001 and score ≥ 3: HR 4.813, 95% CI 3.022–7.668, *P* < 0.001), total gastrectomy (HR 1.620, 95% CI 1.195–2.197, *P* = 0.002) and the presence of postoperative complications (HR 1.402, 95% CI 1.024–1.919, *P* = 0.035) were significant independent risk factors for DOD after gastrectomy for GC, in addition to age of 70 years or higher, performance status of one or higher and body mass index less than 22.0 at gastrectomy.

**Conclusions:**

Pneumonia is a leading cause of DOD after curative gastrectomy and lymph node dissection for GC. Paying attention to comorbidities, minimizing the choice of total gastrectomy and avoiding postoperative complications are essential to maintain the long-term prognosis after gastrectomy.

**Supplementary Information:**

The online version contains supplementary material available at 10.1186/s12893-024-02313-6.

## Background

Gastric cancer (GC) is the world’s fourth leading cause of cancer death [1]. However, over the past 30 years, outcomes after gastrectomy with lymph node dissection, which is the only curative treatment for GC, have been improving [2, 3]. Standardization, surgical treatment centralization and perioperative management development are recognized as contributing factors behind this trend [2, 3]. Furthermore, recent advances in perioperative chemotherapy are expected to bring a cure to more gastric cancer patients [4–6]. Therefore, maintaining patients’ quality of life after curing GC has become an important issue.

One of the subsequent concerns for patients cured of GC is death from other diseases (DOD). Since elderly patients are more likely to have severe comorbidities and physiological dysfunction in major organs [7, 8], they are definitely at high risk of DOD. In addition, the proportion of elderly patients with GC is increasing with the aging of populations [9, 10]. Therefore, clinicians need to pay more attention to DOD in patients with GC. However, the risk factors for DOD after gastrectomy with lymph node dissection for GC have not been elucidated.

In this study, we conducted a prognostic survey of patients undergoing curative gastrectomy with lymph node dissection for GC and investigated the cause of DOD and the risk factors for DOD after gastrectomy, focusing on the patient’s comorbidities and perioperative factors.

## Methods

### Patients

**Fig. 1:**
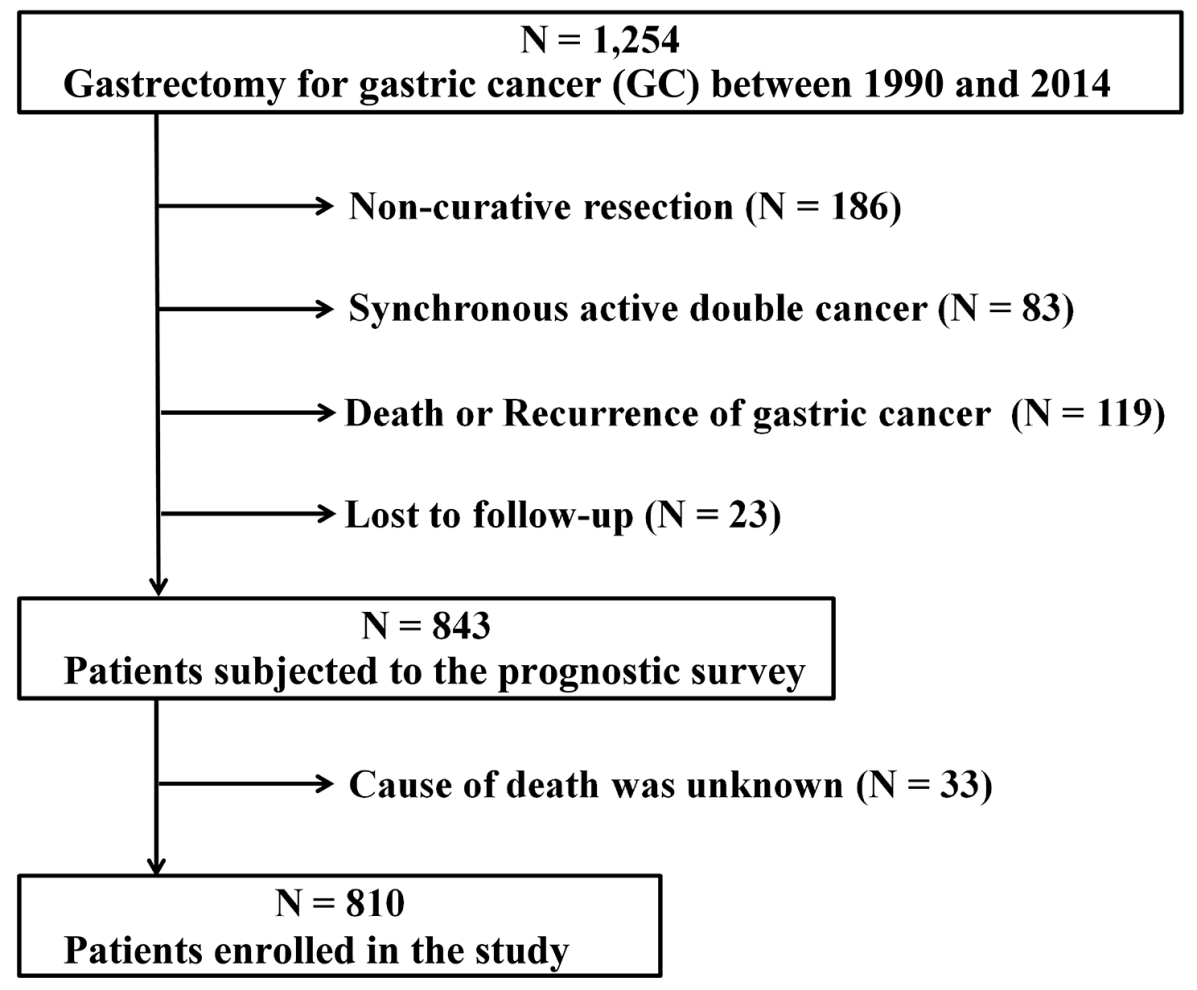
Flowchart of patient enrollment

From a prospectively maintained database in our institution, we enrolled 1,254 consecutive patients with primary GC who had undergone a gastrectomy between January 1990 and December 2014 at the Division of Digestive and General Surgery, Niigata University Medical and Dental Hospital. The exclusion criteria were (1) noncurative resection at the primary surgery (*N* = 186), (2) synchronous active double cancer (*N* = 83), (3) death or recurrence of GC until December 2019 (*N* = 119) and (4) loss to follow-up (*N* = 23). After the exclusion of another 33 patients whose cause of death was unknown at the prognostic survey, a total of 810 patients were enrolled in this study (Fig. 1). This study was approved by the Ethics Committee of the Institutional Review Board (IRB) of Niigata University (No. 2018 − 0137) in accordance with the Ethical Guidelines for Medical and Biological Research Involving Human Subjects in Japan [11]. The need for written informed consent was waived by the IRB because of the retrospective observational nature of the study.

### Comorbidities and postoperative complications

The patient’s comorbidities were assessed on the basis of their medical history, current presentation and examination findings before gastrectomy. We classified the comorbidities into six categories based on the Charlson Comorbidity Index (CCI) scores [12]: (1) cardiovascular disease, (2) respiratory disease, (3) renal disease, (4) liver disease, (5) endocrine and collagen disease and (6) neurological disease (Supplementary Table 1). Postoperative complications after gastrectomy were defined as grade II or higher complications according to the Clavien‒Dindo (CD) classification system [13].

### Tumor characteristics, surgical procedures and follow-up

The characteristics of the primary tumor are described according to the Japanese Classification of Gastric Carcinoma [14]. The type and extent of gastrectomy were selected for each period according to the Japanese Gastric Cancer Treatment Guidelines [15–17]. In general, patients with tumor of the middle or lower third of the stomach underwent distal gastrectomy (DG), and patients with tumor of the upper third of the stomach underwent total gastrectomy (TG). Limited resection, including pylorus-preserving gastrectomy (PPG) and proximal gastrectomy (PG), was also performed at the surgeon’s discretion. The extent of lymph node dissection was determined by the type of gastrectomy. The 810 patients were routinely followed up at our outpatient clinic or affiliated hospitals. A prognostic survey of 507 patients who had stopped visiting our hospital was conducted by the telephone surveys to each affiliated hospitals to collect the prognostic information clinically obtained during a regular follow-up. As of 31 December 2019, 315 of the 810 patients had died from any cause. The median postoperative follow-up time in the survivors was 140 months (range: 60–358 months).

### Definition of DOD and life expectancy at gastrectomy

In this study, DOD was defined as death excluding death from a malignant neoplasm, accident or suicide, which were competing risks. The survival period was from the date of gastrectomy to DOD or the last follow-up. We compared the actual survival period of patients who died from other diseases with their life expectancy to evaluate the influence of gastrectomy on prognosis regarding DOD. The life expectancy for each patient was estimated based on an abridged life table for the year in which the gastrectomy was performed. The abridged life tables were published by the Ministry of Health, Labor and Welfare in Japan and are available from https://www.mhlw.go.jp/toukei/saikin/hw/seimei/list54-57-02.html.

The discrepancy between the actual survival period after gastrectomy and the life expectancy at gastrectomy was calculated according to the following formula: Discrepancy = actual survival period (years) – life expectancy (years).

### Survival analyses

The cumulative incident function was used to calculate the incidence rate of DOD considering the impact of competing risks. We assessed the associations between the incidence of DOD and the following clinicopathological and operative factors: age (< 70 years vs. ≥ 70 years), sex (male vs. female), performance status (PS, 0 vs. 1/2/3), body mass index (BMI, < 22.0 vs. ≥ 22.0), CCI score (0 vs. 1/2 vs. ≥ 3), type of gastrectomy (TG vs. DG/PG/PPG), lymphadenectomy (D1/1 + vs. D2/D3), combined resection (absent vs. present), postoperative stay in the hospital (< 20 days vs. ≥ 20 days), operative time (< 250 min. vs. ≥ 250 min.), blood loss (< 250 mL vs. ≥ 250 mL), postoperative complications (absent vs. present) and pathological stage (pStage I vs. pStage II/III). The Gray test was used as a univariate analysis to assess the differences between the patients’ incidence curves of DOD. Independent prognostic factors were identified using the Fine & Gray subdistribution proportional model as a multivariate analysis: stepwise selection was used for variable selection. All statistical analyses were performed with EZR, which is a modified version of R commander, installed in the R programming language and environment (version 3.5.2; http://www.r-project.org/) [18]. *P* values less than 0.05 were considered statistically significant.

## Results

### Patient characteristics

The clinicopathological and operative characteristics of enrolled patients at gastrectomy for GC are summarized in Table 1. Among the 810 patients enrolled, 587 (72.5%) were male and 223 (27.5%) were female, with a median age of 65 years (range 28–91 years). PS was zero for 679 patients (83.8%), and the median BMI was 22.7 (range 13.1–53.3) before gastrectomy. CCI scores were zero for 542 patients (66.9%) and three or more for 36 (4.4%). Types of gastrectomy were TG for 225 patients (27.8%), DG for 482 (59.5%), PG for 76 (9.4%) and PPG for 27 (3.3%). D2 lymphadenectomy and combined resection of other organs at gastrectomy were performed in 347 (42.8%) and 110 patients (13.6%), respectively. The median operative time, blood loss and postoperative stay in the hospital were 262 min (range 83–724 min), 240 ml (range 5–9580 ml) and 18 days (range 8–325 days), respectively. Regarding postoperative complications, 208 patients (25.7%) had grade II or higher complications after gastrectomy. Pathological stages were pStage I for 661 patients (81.6%), pStage II for 102 patients (12.6%) and pStage III for 47 patients (5.8%).

**Table 1:** Clinicopathological and operative characteristics of enrolled patients

Variables	All patients (*N* = 810)
Age (years)	
Median (range)	65 (28–91)
Sex, N (%)	
Male	587 (72.5)
Female	223 (27.5)
Performance status (ECOG), N (%)	
0	679 (83.8)
1	117 (14.5)
2	10 (1.2)
3	4 (0.5)
Body mass index (kg/m^2^)	
Median (range)	22.7 (13.1–53.3)
Charlson Comorbidity Index score, N (%)	
0	542 (66.9)
1	170 (21.0)
2	62 (7.7)
≥ 3	36 (4.4)
Type of gastrectomy, N (%)	
TG	225 (27.8)
DG	482 (59.5)
PG	76 (9.4)
PPG	27 (3.3)
Lymphadenectomy, N (%)	
D1	300 (37.1)
D1+	146 (18.0)
D2	347 (42.8)
D3	17 (2.1)
Combined resection, N (%)	
Absent	110 (13.6)
Present	700 (86.4)
Postoperative hospital stay (days)	
Median (range)	18 (8–325)
Operative time (min.)	
Median (range)	262 (83–724)
Blood loss (mL)	
Median (range)	240 (5–9580)
Postoperative complications*, N (%)	
Absent	602 (74.3)
Present	208 (25.7)
Pathological stage†, N (%)	
pStage I	661 (81.6)
pStage II	102 (12.6)
pStage III	47 (5.8)

*Grade II or higher according to the Clavien‒Dindo classification system

†Japanese classification of gastric carcinoma: 3rd English edition

TG, total gastrectomy; DG, distal gastrectomy; PG, proximal gastrectomy; PPG, pylorus-preserving gastrectomy

### Causes of death after gastrectomy for gastric cancer

**Fig. 2:**
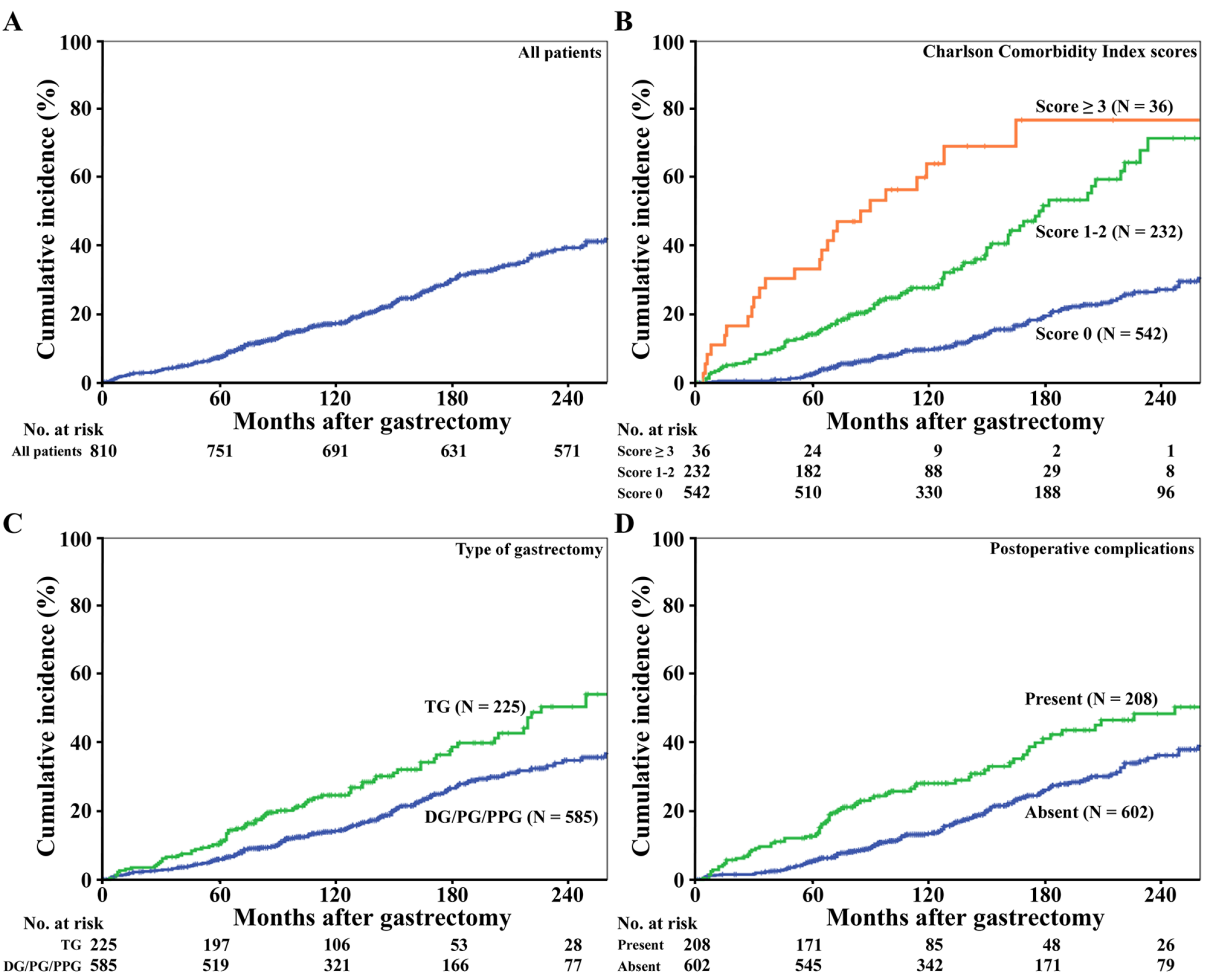
Cumulative incident function plots of the incidence rate of death from other diseases after curative gastrectomy with lymph node dissection for GC in all patients (**A**) and those stratified by the Charlson Comorbidity Index scores (**B**), type of gastrectomy (**C**) and postoperative complications (**D**)

**Fig. 3:**
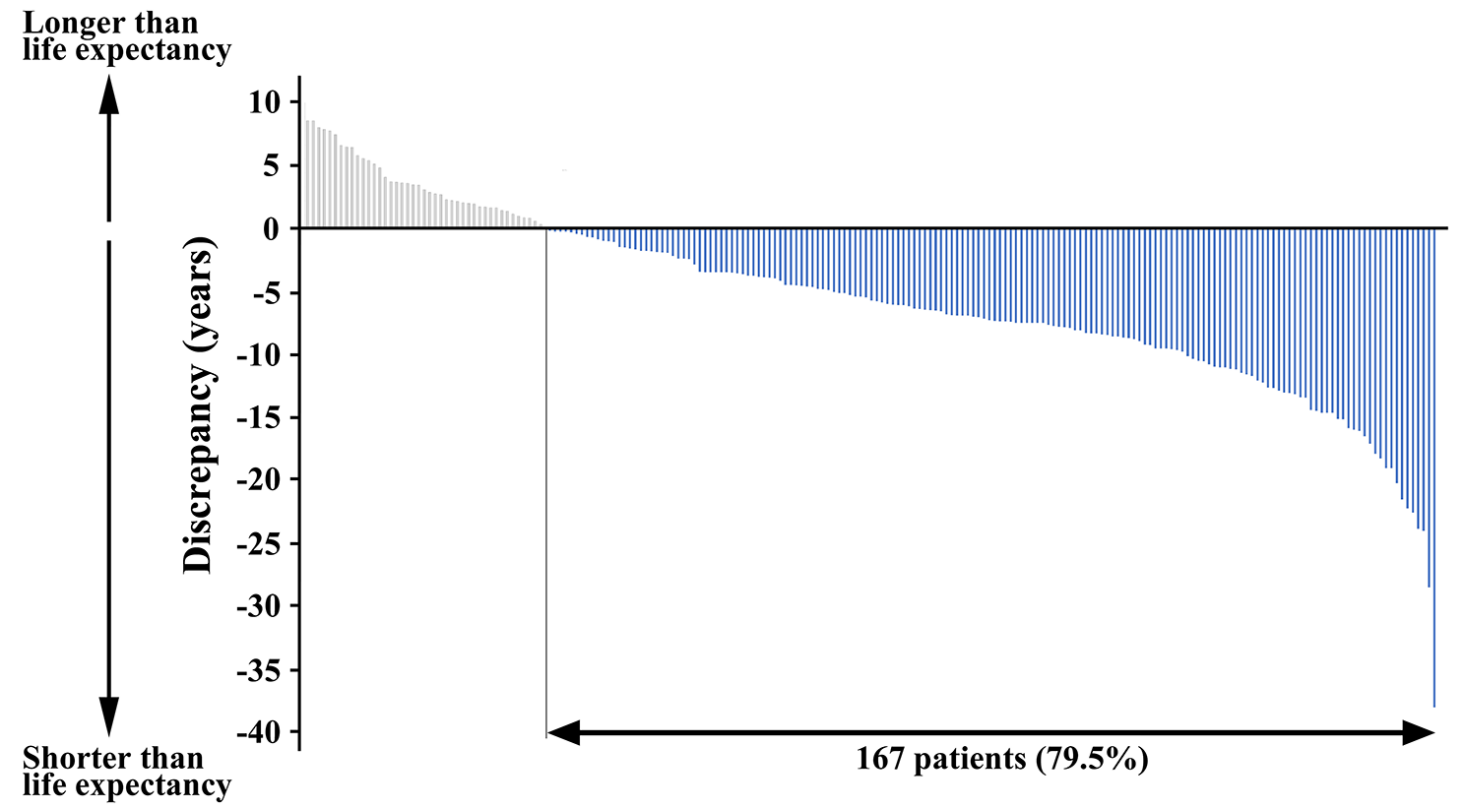
Discrepancy between the actual survival period and the life expectancy in patients with death from other diseases. The actual survival period was compared with the life expectancy expected at gastrectomy according to the abridged life table (*N* = 210)

Among the 315 deaths from any cause, 88 (27.9%) and 17 (5.4%) were deaths from a malignant neoplasm and from accident or suicide, respectively. The remaining 210 deaths (66.7%) were DOD, which were the events in the following survival analyses for the cumulative incidence of DOD. The 5-year/10-year incidence rates of DOD in the enrolled 810 patients were 7.2%/17.2% (Fig. 2A). The leading cause of DOD was pneumonia in 54 patients (25.7%), followed by cardiovascular disease in 37 patients (17.6%); senility, so-called spontaneous death with no other cause of death described, in 36 patients (17.1%); and cerebrovascular disease in 33 patients (15.7%), as detailed in Table 2. The actual survival period in 167 of 210 patients (79.5%) with DOD was shorter than their life expectancy estimated at gastrectomy according to the abridged life table (Fig. 3).

**Table 2:** Causes of death from other diseases in 210 patients

Cause of death	Number of patients (%)
Pneumonia	54 (25.7)
Cardiovascular disease	37 (17.6)
Senility	36 (17.1)
Cerebrovascular disease	33 (15.7)
Pulmonary disease	15 (7.1)
Renal failure	13 (6.2)
Cirrhosis/liver failure	7 (3.3)
Ileus/digestive disease	6 (2.9)
Collagen and metabolic disease	5 (2.4)
Sepsis	4 (1.9)

### Risk factors for death from other diseases after gastrectomy

We evaluated the prognostic impact of clinicopathological and operative factors at gastrectomy on DOD in patients after curative gastrectomy with lymph node dissection for GC. The 5-year/10-year incidence rates of DOD were 2.5%/9.3% in patients with a CCI score of 0, 13.8%/25.8% in those with a CCI score of 1–2 and 33.3%/64.1% in those with CCI score ≥ 3 (*P* < 0.001, Fig. 2B). Patients who underwent TG had a significantly higher incidence of DOD than those who underwent other types of gastrectomy (5-year/10-year, 10.2%/24.1% vs. 5.8%/13.6%, *P* < 0.001, Fig. 2C). The presence of postoperative complications of CD grade II or higher was significantly associated with a high incidence of DOD (5-year/10-year, 12.5%/27.2% vs. 5.2%/12.9%, *P* < 0.001, Fig. 2D). In addition, the univariate analysis revealed that age (*P* < 0.001), PS (*P* < 0.001), BMI (*P* = 0.019), lymphadenectomy (*P* < 0.001), postoperative hospital stay (*P* = 0.001), operative time (*P* = 0.044) and pathological tumor stage (*P* = 0.002) were significant risk factors for DOD after gastrectomy (Table 3). We entered the 10 significant factors in univariate analyses into the Fine & Gray subdistribution proportional model as a multivariate analysis. The results showed that a high CCI score (score 1–2: hazard ratio [HR] 2.192, 95% confidence interval [CI] 1.713–2.804, *P* < 0.001 and score ≥ 3: HR 4.813, 95% CI 3.022–7.668, *P* < 0.001), TG (HR 1.620, 95% CI 1.195–2.197, *P* = 0.002) and the presence of postoperative complications (HR 1.402, 95% CI 1.024–1.919, *P* = 0.035) were significant independent risk factors for DOD after gastrectomy, in addition to age of 70 years or higher, PS of one or higher and BMI less than 22.0 at gastrectomy (Table 4).

**Table 3:** Univariate analysis of risk factors for death from other diseases

Variables	Number of patients	Incidence rate of DOD (%)		*P* value
		5-year	10-year	
Age (years)				< 0.001
< 70	529	2.6	8.2	
≥ 70	281	15.3	32.5	
Gender				0.233
Male	587	7.5	17.9	
Female	223	5.8	12.8	
Performance status (ECOG)				< 0.001
0	679	3.8	10.9	
1/2/3	131	23.7	44.6	
Body mass index (kg/m^2^)				0.019
< 22.0	334	8.7	21.3	
≥ 22.0	476	5.9	13.1	
Charlson Comorbidity Index score				< 0.001
0	542	2.5	9.3	
1/2	232	13.8	25.8	
≥ 3	36	33.3	64.1	
Cardiovascular disease				< 0.001
Absent	749	6.4	15.3	
Present	61	14.8	32.0	
Respiratory disease				< 0.001
Absent	756	6.1	15.0	
Present	54	20.4	38.4	
Renal disease				< 0.001
Absent	775	5.5	14.3	
Present	35	40.0	63.1	
Liver disease				0.033
Absent	775	6.5	15.8	
Present	35	20.0	31.7	
Endocrine and collagen disease				0.006
Absent	711	6.2	15.3	
Present	99	13.1	25.1	
Neurological disease				< 0.001
Absent	759	6.2	15.2	
Present	51	19.6	37.5	
Type of gastrectomy				< 0.001
TG	225	10.2	24.1	
DG/PG/PPG	585	5.8	13.6	
Lymphadenectomy				< 0.001
D1/1+	446	10.3	22.0	
D2/3	364	3.0	10.0	
Combined resection				0.122
Absent	700	7.4	16.4	
Present	110	4.5	16.7	
Postoperative stay in hospital (days)				0.001
< 20	450	5.3	12.3	
≥ 20	360	9.2	21.5	
Operative time (min.)				0.044
< 250	337	11.0	21.0	
≥ 250	473	4.2	13.2	
Blood loss (mL)				0.813
< 250	422	7.6	15.6	
≥ 250	388	6.4	17.1	
Postoperative complications*				< 0.001
Absent	602	5.2	12.9	
Present	208	12.5	27.2	
Pathological stage†				0.002
pStage I	661	5.4	14.3	
pStage II/III	149	14.1	26.1	

*Grade II or higher according to the Clavien‒Dindo classification system

†Japanese classification of gastric carcinoma: 3rd English edition

DOD, death from other diseases; TG, total gastrectomy; DG, distal gastrectomy; PG, proximal gastrectomy; PPG, pylorus preserving gastrectomy

**Table 4:** Multivariate analysis of risk factors for death from other diseases

Variables	Hazard ratio	95% confidence interval	*P* value
Age (years)			
< 70	1.000		
≥ 70	3.013	2.187–4.151	< 0.001
Performance status (ECOG)			
0	1.000		
1/2/3	1.609	1.086–2.384	0.018
Body mass index (kg/m^2^)			
< 22.0	1.384	1.041–1.839	0.025
≥ 22.0	1.000		
Charlson Comorbidity Index score			
0	1.000		
1/2	2.192	1.713–2.804	< 0.001
≥ 3	4.813	3.022–7.668	< 0.001
Type of gastrectomy			
TG	1.620	1.195–2.197	0.002
DG/PG/PPG	1.000		
Postoperative complications*			
Absent	1.000		
Present	1.402	1.024–1.919	0.035

*Grade II or higher according to the Clavien‒Dindo classification system

TG, total gastrectomy; DG, distal gastrectomy; PG, proximal gastrectomy; PPG, pylorus preserving gastrectomy

Finally, we assessed the influence of each comorbid condition on DOD after gastrectomy for GC using the Fine & Gray subdistribution proportional model (Table 5). The HR values for DOD were 1.992 for cardiovascular disease (*P* < 0.001), 2.167 for respiratory disease (*P* < 0.001), 4.647 for renal disease (*P* < 0.001) and 2.320 for liver disease (*P* = 0.003).

**Table 5:** Hazard ratio for death from other diseases according to comorbidity

Variables	Hazard ratio	95% confidence interval	*P* value
Cardiovascular disease			
Absent	1.000		
Present	1.992	1.392–2.849	< 0.001
Respiratory disease			
Absent	1.000		
Present	2.167	1.446–3.248	< 0.001
Renal disease			
Absent	1.000		
Present	4.647	2.920–7.396	< 0.001
Liver disease			
Absent	1.000		
Present	2.320	1.320–4.078	0.003
Endocrine and collagen disease			
Absent	1.000		
Present	1.084	0.693–1.695	0.720

## Discussion

The average life expectancy has been markedly increasing worldwide, owing to progress against many diseases and injuries [19]. In addition, current advances in curative treatment for GC have allowed an increasing proportion of patients to be cured of GC. Therefore, improving the long-term prognosis after GC cure has become an important issue for patients who undergo curative treatment. In this study, we conducted a prognostic survey of patients who achieved long-term survival after curative gastrectomy with lymph node dissection to elucidate the cause of DOD after gastrectomy and to identify the risk factors for it focusing on the patient’s comorbidities and perioperative factors. As a result, pneumonia was identified as a leading cause of DOD, and to the best of our knowledge, we first demonstrated that 79.5% of patients with DOD could not survive longer than their estimated life expectancy at gastrectomy. High CCI score at gastrectomy, TG and the presence of postoperative complications were shown to be significant independent risk factors for DOD after gastrectomy for GC.

Pneumonia and aspiration pneumonia are currently the fifth and sixth leading cause of death in Japan, respectively [20]. According to the vital statistics in Japan [20], the proportion of pneumonia including aspiration pneumonia is estimated to be 12.3% among the causes of death in the Japanese general population, excluding death from a malignant neoplasm, accident or suicide (Supplementary Table 2). In this study, pneumonia was revealed to be a leading cause of DOD after gastrectomy for GC with 25.7% among the causes of DOD. The development of pneumonia in patients who have undergone gastrectomy seems to be more frequent than in the general population. In addition, the majority of patients with DOD after gastrectomy are not able to survive longer than their estimated life expectancy at gastrectomy. Therefore, gastrectomy might confer an unfavorable prognosis regarding DOD. After surgical treatment for GC, sarcopenia, characterized by progressive and generalized loss of skeletal muscle mass and strength [21], is likely to occur due to surgical stress and nutritional disorders. Postoperative sarcopenia has been reported to be associated with postoperative complications and to result in an unfavorable prognosis after gastrectomy [22]. The progression of sarcopenia may lead to a loss of swallowing-related muscles and eventually to dysphagia, resulting in aspiration pneumonia. In addition, postoperative reflux causing reflux esophagitis, one of the major postoperative problems [23, 24], may also have a deleterious effect on death from pneumonia after gastrectomy. Moreover, our present analyses demonstrated that a preoperative low BMI was an independent risk factor for DOD. Lee et al. [25] also examined the association between BMI and prognosis in patients with GC, and they reported that overall survival was poor in low-weight patients. In patients with GC, nutritional management and the prevention of sarcopenia are particularly important to reduce the risk of DOD, including pneumonia.

The incidence of GC located in the upper third of the stomach is increasing steadily over time [26]. The standard procedure for upper GC is a TG, but TG has been reported to increase the likelihood of complications and cause weight loss and malnutrition, and these factors have been reported to be poor prognostic factors after surgical treatment in patients with GC [27–33]. In particular, TG for elderly patients is more likely to cause weight loss and malnutrition due to reduced oral intake compared to that of younger patients. Our present prognostic survey also revealed that TG was one of the independent risk factors for DOD. Although multiple factors may be involved — such as complications associated with TG and postoperative malnutrition — it is important to avoid performing TG as much as possible, especially when selecting the surgical procedure for elderly patients.

Because the incidence of chronic disease increases with aging, elderly patients are likely to have more severe comorbidities than younger patients. As the proportion of elderly patients with GC is increasing [9, 10], preoperative assessment of the severity of comorbidities is essential not only for the short-term outcome but also for maintaining the long-term prognosis of patients who undergo surgical treatment for GC. A previous study demonstrated that preoperative comorbidities affected not only the development of perioperative complications and mortality but also the long-term prognosis after gastrectomy [34]. The CCI was developed as a predictor of 1-year mortality in hospitalized patients and of 10-year survival in breast cancer [12], and it has been widely used to assess the severity of comorbidities in patients with solid tumors. Our present study revealed that the CCI was also an independent risk factor for DOD and was useful as an index for objectively scoring preoperative comorbidities. The results of our analysis of comorbidities classified into six categories suggested that patients with respiratory diseases or renal diseases were at higher risk of DOD over the long postgastrectomy period than patients with other comorbidities. Patients who had a high CCI with these comorbidities should be carefully followed for a long period of time, considering the high risk of DOD after gastrectomy for GC.

Several studies have shown that postoperative complications adversely affect the oncological prognosis in various cancers, including GC [32, 34–38]. It was recently suggested that immunosuppression and inflammatory cytokines induced by inflammatory responses may promote the metastasis of floating cancer cells and residual microscopic disease [39]. We found that the presence of postoperative complications was an independent risk factor for DOD after gastrectomy. Postoperative complications prolong the length of a patient’s hospital stay, exacerbate any postoperative disability, and reduce the patient’s activities of daily living. Weight loss and malnutrition are likely to occur after gastrectomy, and these physical deficits may continue to negatively affect the patient’s prognosis for a long time after gastrectomy. Preventing postoperative complications not only improves cancer-specific survival but also contributes to a favorable long-term prognosis regarding DOD after gastrectomy.

This study has some limitations to consider. The study design was retrospective and single-institutional, with patients drawn from a long period of time. There may have been a selection bias since the surgical selection criteria could have changed over time, and the treatment modifications depended on the clinicians’ discretion based on the general condition of each patient. Because laparoscopic gastrectomy, which is an acceptable procedure for GC currently, has not been performed as a standard procedure during the period of the inclusion criteria, a minority of patients with highly biased backgrounds underwent laparoscopic gastrectomy. Thus, we could not investigate the association between laparoscopic gastrectomy and DOD after gastrectomy in this study. In addition, with medical advances, patients’ life expectancy has gradually increased over time, and it is thus unclear whether our present findings can be generalized. However, this study was based on extremely valuable prognostic data with a thorough prognostic survey for patients after GC treatment. We believe that the findings of this study provide useful information for considering surgical strategies and patient follow-up in the era of further improvement in GC treatment outcomes.

## Conclusions

Comorbidities, total gastrectomy and postoperative complications can lead to a long-term risk of DOD in patients who underwent curative gastrectomy with lymph node dissection for GC. Clinicians should therefore make appropriate surgical decisions based on the assessment of each patient’s comorbidities, avoiding total gastrectomy when possible and performing a reliable surgery without postoperative complications.

### Electronic supplementary material

Below is the link to the electronic supplementary material.

Supplementary Material 1

Supplementary Material 2

## Data Availability

All data generated or analyzed during this study are included in this article. Further inquiries can be directed to the corresponding author.

## Abbreviations

GC: Gastric cancer
DOD: Death from other diseases
IRB: Institutional review board
CCI: Charlson comorbidity index
CD: Clavien‒Dindo
DG: Distal gastrectomy
TG: Total gastrectomy
PPG: Pylorus-preserving gastrectomy
PG: Proximal gastrectomy
PS: Performance status
BMI: Body mass index
HR: Hazard ratio
CI: Confidence interval
