# Supplementary material for: Risk factors for death from other diseases after curative gastrectomy and lymph node dissection for gastric cancer
Source: BMC Surg. 2024 Jan 8;24:16. doi: 10.1186/s12893-024-02313-6 (PMC10775521; doi:10.1186/s12893-024-02313-6)
Supplement: Supplementary file 1 — Supplementary Material 1 [file 12893_2024_2313_MOESM1_ESM.doc]

| **Supplementary Table 1.** Classification of comorbidities based on the Charlson Comorbidity Index scores | |
| --- | --- |
| Comorbidities | Score |
| Cardiovascular disease |  |
| Myocardial infarction | 1 |
| Congestive heart failure | 1 |
| Peripheral vascular disease | 1 |
| Respiratory disease |  |
| Chronic pulmonary disease | 1 |
| Renal disease |  |
| Moderate or severe renal disease | 2 |
| Liver disease |  |
| Mild liver disease | 1 |
| Moderate or severe liver disease | 3 |
| Endocrine and collagen disease |  |
| Connective tissue disease | 1 |
| Diabetes (excludes diet-controlled alone) | 1 |
| Diabetes with end-organ damage | 2 |

| **Supplementary Table 1.** (continued) |  |
| --- | --- |
| Comorbidities | Score |
| Neurological disease |  |
| Cerebrovascular disease | 1 |
| Dementia | 1 |
| Hemiplegia or paraplegia | 2 |
|  |  |
