# Supplementary material for: Risk factors for death from other diseases after curative gastrectomy and lymph node dissection for gastric cancer
Source: BMC Surg. 2024 Jan 8;24:16. doi: 10.1186/s12893-024-02313-6 (PMC10775521; doi:10.1186/s12893-024-02313-6)
Supplement: Supplementary file 2 — Supplementary Material 2 [file 12893_2024_2313_MOESM2_ESM.doc]

| **Supplementary Table 2.** Mortality rate in the Japanese general population in 2021 | | |
| --- | --- | --- |
| Cause of death* | Mortality rate (per 100 000 population) | Proportion (%)‡ |
| Malignant neoplasms | 310.7 | - |
| Cardiovascular disease | 174.9 | 21.6 |
| Senility | 123.8 | 15.3 |
| Cerebrovascular disease | 85.2 | 10.5 |
| Pneumonia | 59.6 | 7.3 |
| Aspiration pneumonia | 40.3 | 5.0 |
| **Pneumonia and aspiration pneumonia** | **99.9** | **12.3** |
| Accident† | 34.1 | - |
| Suicide | 16.5 | - |
| All deaths | 1172.7 | - |
| Deaths excluding malignant neoplasms, accident and suicide | 811.4 | - |
| * First to sixth leading causes of deaths, accidents and suicide are presented in the table.  † Accident includes the traffic accident (2.9 per 100 000 population).  ‡ Among the deaths excluding malignant neoplasms, accident and suicide. | | |
